# Fractal analysis of peri-implant trabecular bone complexity following transcrestal sinus floor elevation: a retrospective radiographic study

**DOI:** 10.3389/fmed.2026.1943053

**Published:** 2026-09-03

**Authors:** Irfan Ustundag, Sevval Ilkyaz, Numan Dedeoglu, Selman Bolukbasi, Mehmet Sait Simsek, Yunus Çetiner, Bahadir Sancar

**Affiliations:** 1 Department of Oral and Maxillofacial Surgery, Faculty of Dentistry, Inonu University, Malatya, Turkiye; 2 Department of Oral and Maxillofacial Radiology, Faculty of Dentistry, Inonu University, Malatya, Turkiye; 3 Faculty of Health Sciences, Department of Gerontology, Balikesir University, Balikesir, Turkiye; 4 Department of Oral and Maxillofacial Surgery, Faculty of Dentistry, Adiyaman University, Adiyaman, Turkiye

**Keywords:** dental implant, fractal analysis, panoramic radiography, trabecular bone, transcrestal sinus floor elevation

## Abstract

**Background:**

This retrospective radiographic study aimed to evaluate time- and region-dependent changes in peri-implant trabecular bone structural complexity using fractal dimension analysis after transcrestal sinus floor elevation (TSFE) with 3, 4, or 5 mm sinus membrane elevation.

**Methods:**

A total of 45 dental implants placed simultaneously with TSFE in 35 patients were categorized into three groups according to sinus membrane elevation height (3 mm, 4 mm, and 5 mm). Fractal dimension analysis was performed using ImageJ software on panoramic radiographs obtained at baseline and at 6 and 12 months postoperatively, in the apical, mesial, and distal peri-implant regions.

**Results:**

No statistically significant difference was found among the 3 mm, 4 mm, and 5 mm groups in fractal dimension changes from baseline to 6 or 12 months postoperatively (*P* > 0.05). In the 4 mm and 5 mm groups, no statistically significant temporal FD changes were detected in any region (*P* > 0.05). In the 3 mm group, the distal fractal dimension value at 6 months was significantly lower than the apical value (*P* = 0.006), whereas a significant increase was observed in the distal region between 6 and 12 months (*P* = 0.013).

**Conclusion:**

No statistically significant between-group differences in peri-implant trabecular bone structural complexity were detected among the 3 mm, 4 mm, and 5 mm TSFE groups over 12 months. These preliminary findings do not establish equivalence and require prospective confirmation; the transient distal change in the 3 mm group should be interpreted cautiously as a region-specific, hypothesis-generating observation.

## Graphical Abstract

**Figure:**
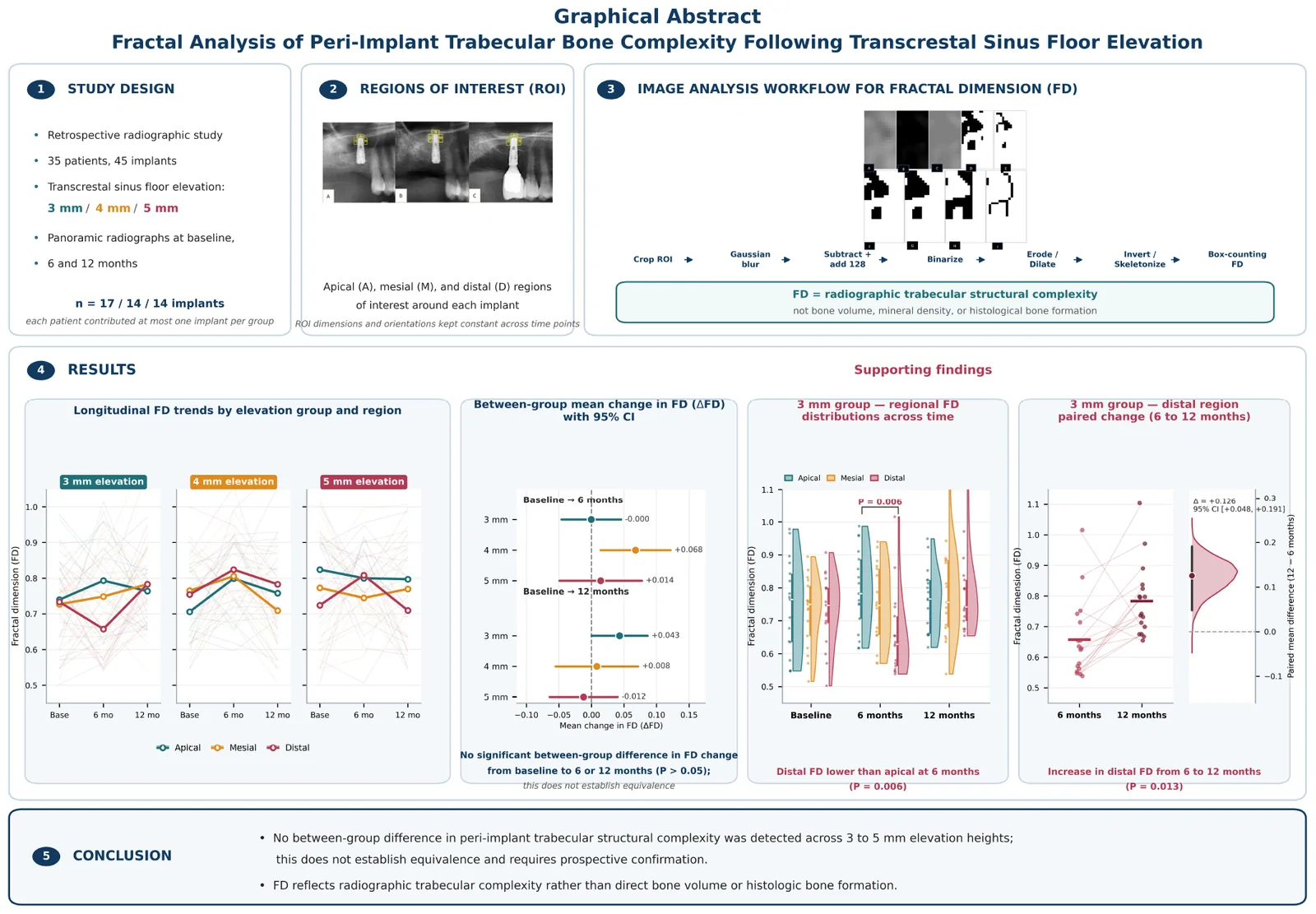
Study design, fractal dimension analysis workflow, and main findings regarding peri-implant trabecular bone following transcrestal sinus floor elevation with 3, 4, or 5 mm of membrane elevation.

## Introduction

In dental implantology, the posterior maxilla is considered one of the most challenging anatomical regions from both surgical and prosthetic perspectives. Implant placement in this region is mainly limited by maxillary sinus pneumatization, vertical resorption of the posterior alveolar bone, and poor bone quality (1, 2). Following tooth loss, alveolar bone loss and inferior expansion of the sinus floor may further reduce the bone volume required for ideal implant positioning and adequate primary stability (2, 3).

Various maxillary sinus augmentation techniques have been developed to overcome these limitations and enable implant-supported rehabilitation in the posterior maxilla. The sinus floor elevation procedure was first described by Tatum and later became widely used after Boyne and James introduced the lateral window approach into clinical practice (4, 5). Maxillary sinus floor elevation techniques are currently classified into two main approaches: lateral window sinus floor elevation and transcrestal sinus floor elevation (TSFE), the latter also commonly referred to as the closed or internal approach (6).

The TSFE technique described by Summers involves controlled elevation of the sinus floor using osteotomes and atraumatic elevation of the Schneiderian membrane (7). In appropriate cases, it can be performed simultaneously with implant placement and offers advantages over the lateral window approach, including less invasiveness, shorter surgical time, and reduced postoperative morbidity (8). However, because the surgical field cannot be directly visualized and the amount of elevation is limited, careful evaluation is required, particularly in cases requiring greater membrane elevation (9).

The success of TSFE depends on residual bone support, sinus membrane elasticity, elevation height, implant primary stability, and clinician experience (10). Previous studies have reported that TSFE provides limited vertical bone gain and that membrane elevation should be performed in a controlled manner to reduce the risk of perforation (11). Therefore, evaluating peri-implant bone response at different elevation heights is important for understanding the clinical limits of this technique and the dynamics of bone healing.

Conventional radiographs are widely used to evaluate bone healing around dental implants. However, visual radiographic assessment may be insufficient to detect subtle changes in trabecular bone architecture. Accordingly, interest in quantitative radiographic analysis methods has increased. Fractal analysis is a mathematical method used to evaluate the geometric complexity of irregular and self-similar structures (12). Because of its branching and complex architecture, trabecular bone represents a suitable biological model for fractal analysis (13).

Fractal dimension (FD) is a quantitative image-derived parameter that provides indirect information about the radiographic structural organization and complexity of trabecular bone (13). Changes in FD values may reflect alterations in trabecular architecture associated with bone remodeling and peri-implant bone response (14). From a clinical perspective, FD analysis may complement conventional radiographic assessment by quantifying changes in radiographic trabecular structural complexity. However, FD does not directly measure bone mineral density, histological bone volume, load distribution, implant stability, or biomechanical performance; therefore, it should be interpreted specifically as an indicator of radiographic trabecular structural complexity (14).

Although previous studies have evaluated bone changes after open sinus lift procedures, studies investigating peri-implant trabecular bone structural complexity using fractal analysis after TSFE at different membrane elevation heights remain limited (15, 16). Comparing time-dependent FD changes in the apical, mesial, and distal regions among 3 mm, 4 mm, and 5 mm elevation groups may help clarify regional bone healing patterns after TSFE.

The aim of this study was to evaluate peri-implant trabecular bone structural complexity using FD analysis in cases where implants were placed simultaneously with TSFE and the sinus membrane was elevated by 3 mm, 4 mm, or 5 mm. FD measurements were performed in the apical, mesial, and distal regions on baseline panoramic radiographs obtained on the day of implant placement and on postoperative 6- and 12-month panoramic radiographs. Changes were compared according to elevation height and time point.

## Materials and methods

### Study design and ethical approval

Ethical approval was obtained from the institutional Health Sciences Scientific Research and Publication Ethics Committee (Date: 04.02.2025; Decision No: 2025/6702). The requirement for written informed consent was waived by the ethics committee due to the retrospective nature of the study and the use of anonymized radiographic data. The study was conducted in accordance with the principles of the Declaration of Helsinki.

### Study sample

A total of 45 dental implants placed in 35 patients who underwent simultaneous TSFE were included. All surgical procedures were performed by the same surgeon. Demographic data, clinical findings, surgical details, and implant-related information were obtained retrospectively from patient records.

The implants were classified according to sinus membrane elevation height as 3 mm, 4 mm, and 5 mm. For each implant, fractal dimension measurements were performed in three peri-implant regions: apical, mesial, and distal. Radiographic evaluations were performed using panoramic radiographs obtained at baseline and at postoperative 6 and 12 months.

### Inclusion and exclusion criteria

Patients aged between 30 and 65 years who underwent simultaneous implant placement with TSFE in the posterior maxilla, whose sinus membrane was elevated by 3 mm, 4 mm, or 5 mm, and who had complete panoramic radiographs at baseline, 6 months, and 12 months were included. Patients with systemic disease, a history of sinusitis, tobacco use, missing follow-up radiographs, or radiographic images unsuitable for fractal analysis were excluded.

### Power analysis

The sample size was calculated using G*Power version 3.1.9.7. For three groups, the effect size was set at f = 0.60, with alpha = 0.05, beta = 0.20, and a statistical power of 0.80. The analysis indicated that a minimum total sample size of 30, with at least 10 samples per group, was required. The obtained sample of 45 implants is adequate for detecting an effect of this magnitude but may be underpowered for smaller between-group differences. The large effect size was adopted *a priori* because of the feasibility constraints of this retrospective sample and the expectation that any clinically meaningful difference in trabecular structural complexity among the elevation groups would be substantial; this assumption is acknowledged as a limitation, and the non-significant findings are interpreted accordingly.

### Surgical protocol

According to the surgical records, all implant surgeries were performed by the same surgeon under local anesthesia. A mid-crestal incision was made, and a full-thickness mucoperiosteal flap was elevated. The implant site was prepared using drills compatible with the implant diameter, and the osteotomy was terminated approximately 1 mm short of the sinus floor. The Schneiderian membrane was carefully elevated using osteotomes. Because of the retrospective design, sinus membrane integrity was determined from the contemporaneous operative records. Membrane integrity was documented as having been maintained in all procedures, and no sinus membrane perforation was recorded. Sinus membrane elevation height was determined using both the contemporaneous surgical records and radiographic measurements. The elevation height documented in the surgical records was expressed in millimeters. To verify this information radiographically, the baseline panoramic image was calibrated using the known actual implant length, and the distance between the original maxillary sinus floor and the implant apex was measured parallel to the long axis of the implant. The surgical records and radiographic measurements were cross-checked, and the implants were accordingly classified into the 3 mm, 4 mm, and 5 mm elevation groups. The radiographic measurement was used to verify the recorded elevation category rather than as a direct measurement of the elevated membrane contour. No bone graft or other grafting material was used in any of the procedures. Dental implants were placed during the same surgical session, and the flap was primarily closed with sutures. Standard postoperative medical treatment and oral hygiene instructions were provided. Definitive prosthetic loading was performed between 6 and 9 months after implant placement in all cases.

### Radiographic evaluation

All panoramic radiographs were acquired using a Planmeca ProMax 2D panoramic X-ray unit (Planmeca Oy, Helsinki, Finland) with exposure parameters of 66 kVp, 8 mA, and 15.8 s and a total filtration of 2.5 mm Al. The image matrix was 1962 × 958 pixels, and all images were evaluated using Romexis software (Planmeca Oy, Helsinki, Finland). Only radiographs obtained in accordance with the manufacturer’s standardized patient-positioning protocol were included. During image acquisition, the patient’s midsagittal plane was aligned with the device’s midline laser beam and positioned perpendicular to the floor, the Frankfort horizontal plane was positioned parallel to the floor, and the upper and lower incisors were placed in the designated groove of the bite block to position the dental arch within the focal trough. Radiographs showing motion artifacts, positioning errors, marked geometric distortion, or metal artifacts affecting the evaluated peri-implant regions were excluded. Image-processing settings were maintained consistently across all panoramic radiographs. Panoramic radiographs obtained on the day of implant placement were accepted as baseline images, and radiographs obtained at postoperative 6 and 12 months were used as follow-up images. Thus, each implant was evaluated at three time points: baseline, 6 months, and 12 months.

Residual bone height was measured on the baseline panoramic radiograph using the linear measurement tool in Romexis software. At the implant site, the distance between the alveolar crest and the original floor of the maxillary sinus was measured parallel to the long axis of the implant and recorded in millimeters. Before measurement, each panoramic image was calibrated using the known actual length of the implant as the reference. All RBH measurements were performed by the same examiner using a standardized measurement protocol.

**FIGURE 1:**
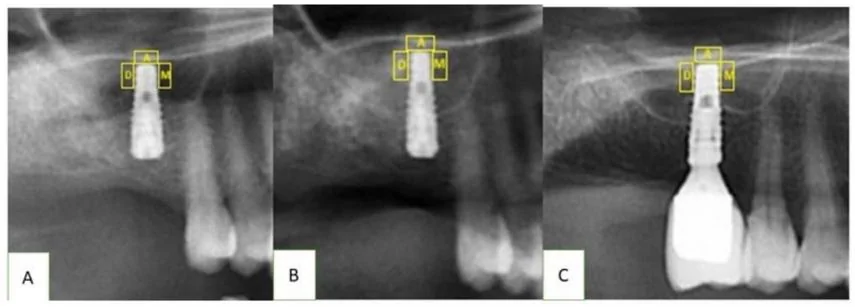
Representative placement of the regions of interest on cropped panoramic radiographs. **(A–C)** show the baseline, postoperative 6-month, and postoperative 12-month radiographs, respectively. The ROI labels A, M, and D denote the apical, mesial, and distal regions, respectively. The implant long axis, apex, and mesial and distal surfaces were used as fixed reference landmarks to reproduce ROI placement across the three time points. ROI dimensions and orientations were maintained unchanged.

For fractal analysis, three regions of interest (ROIs) were defined around each implant. The apical ROI measured 20 × 10 pixels, whereas the mesial and distal ROIs measured 10 × 20 pixels. To standardize ROI placement across the baseline, 6-month, and 12-month radiographs, each implant served as a fixed internal radiographic reference. Following image calibration, the implant long axis, apex, and mesial and distal surfaces were used as reference landmarks. The apical ROI was positioned immediately adjacent to the implant apex, whereas the mesial and distal ROIs were positioned immediately adjacent to the corresponding implant surfaces at the same relative level along the implant body. The baseline ROI positions were manually reproduced on the 6- and 12-month radiographs by matching these implant-related landmarks. ROI dimensions and orientations were maintained consistently across all time points. All ROI selections were performed by the same examiner using a standardized protocol (Figure 1).

### Fractal analysis

Fractal analysis was performed by an experienced researcher using ImageJ software version 1.53 (National Institutes of Health, Bethesda, MD, USA). Before analysis, the panoramic radiographs were converted to TIFF format. Image preprocessing and FD calculation were performed using the modified box-counting method described by White and Rudolph (17, 18).

**FIGURE 2:**
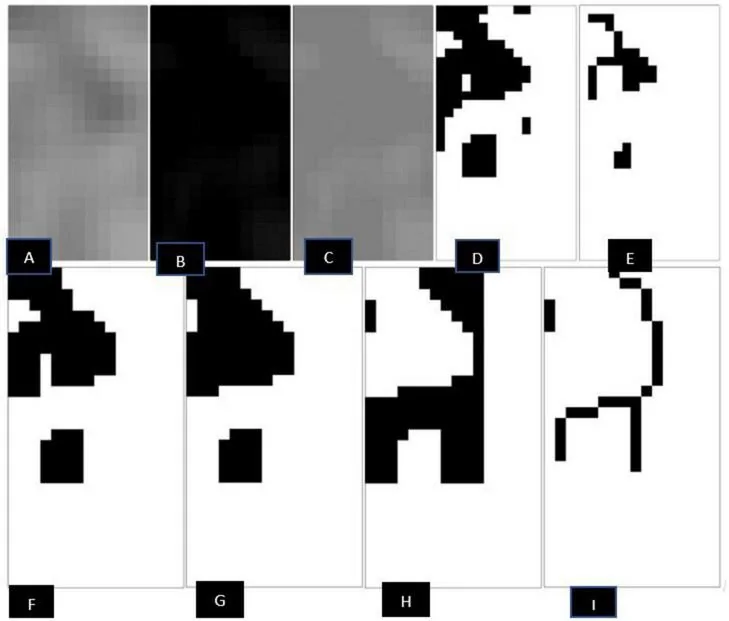
Sequential steps of the fractal dimension analysis workflow: **(A)** Gaussian-blurred duplicate of the cropped ROI; **(B)** subtraction of the blurred duplicate from the original ROI; **(C)** addition of a gray value of 128 to each pixel; **(D)** binarization; **(E)** erosion; **(F)** dilation; **(G)** removal of non-trabecular outliers; **(H)** inversion; and **(I)** skeletonization.

Each selected ROI was converted to 8-bit format and duplicated. Following the White-Rudolph preprocessing protocol, a Gaussian blur filter with a sigma of 35 pixels was applied to each duplicated ROI to generate a low-frequency background image. The same preprocessing parameters were applied consistently to all ROIs and time points. The blurred image was subtracted from the original image, and a gray value of 128 was added to each pixel. The resulting image was then binarized. To reduce noise, the “Erode” and “Dilate” functions were applied. Following erosion and dilation, isolated components comprising fewer than 2 pixels that were not connected to the trabecular network, together with edge artifacts, were removed. The image was then inverted and skeletonized (Figure 2).

Fractal dimension values were calculated using the Fractal Box Count function in ImageJ with square box sizes of 2, 3, 4, 6, 8, 12, 16, 32, and 64 pixels. The linearity of the log-log box-counting regression was assessed using the coefficient of determination (R^2^), with R^2^ ≥ 0.95 considered acceptable. All measurements met this criterion; therefore, no measurement was excluded on the basis of regression fit.

### Measurement reliability

To assess measurement repeatability, the intraclass correlation coefficient (ICC) was calculated with a 95% confidence interval. Panoramic radiographs were randomly selected from each group, and fractal analysis measurements were repeated by the same researcher 2 weeks later. The obtained ICC values were used to evaluate measurement reliability.

### Statistical analysis

The normality of continuous variables was assessed using graphical methods and the Shapiro-Wilk test. Most variables were consistent with a normal distribution and were presented as mean plus/minus standard deviation; a small number of distal-region measurements deviated from normality. One-way ANOVA was used to compare fractal dimension values among the independent 3 mm, 4 mm, and 5 mm groups. Because the apical, mesial, and distal measurements were obtained from the same implant, the regional comparisons at each time point were performed using repeated-measures ANOVA with region as the within-implant factor, and changes over time within each region were likewise evaluated using repeated-measures ANOVA. Because each patient contributed at most one implant to any given elevation group, the within-group regional and temporal analyses involved mutually independent implants; within-patient clustering could therefore affect only the between-group comparisons, for which mixed-effects models were used. Bonferroni correction was applied for pairwise comparisons when significant differences were detected. For the comparisons in which a contributing variable deviated from normality, the corresponding significant results were additionally verified using nonparametric tests, namely the Friedman test for related samples and the Wilcoxon signed-rank test for pairwise comparisons. To account for the non-independence of multiple implants obtained from the same patient, the primary between-group comparisons of fractal dimension change were additionally analyzed using linear mixed-effects models with sinus elevation group as a fixed effect and patient as a random intercept. Because baseline residual bone height differed among the groups, the between-group comparisons were also repeated using analysis of covariance (ANCOVA) and mixed-effects models that included baseline residual bone height as a covariate. To control the false discovery rate across the multiple regional and temporal comparisons, the corresponding *p*-values were additionally adjusted using the Benjamini-Hochberg procedure. To complement null-hypothesis testing, paired mean differences were additionally summarized with bootstrap 95% confidence intervals for the region and interval in which a significant time effect was detected.

All statistical analyses were performed using IBM SPSS Statistics version 26.0 and Microsoft Excel 2024. A *p* value < 0.05 was considered statistically significant. For reliability analysis, 35.2% of the 3 mm group, 29.4% of the 4 mm group, and 29.4% of the 5 mm group were randomly selected, and all measurements were repeated by the same researcher 2 weeks later. This retrospective observational study was reported in accordance with the STROBE guidelines. The study methodology and statistical analysis were reviewed by an independent statistician.

## Results

A total of 45 implants placed in 35 patients were analyzed. Of the 45 implants, 22 were placed in male patients and 23 were placed in female patients. The mean age of the patients was 45.6 ± 9.4 years, with a range of 31 to 63 years. The 3 mm, 4 mm, and 5 mm groups comprised 17, 14, and 14 implants obtained from 17, 14, and 14 patients, respectively; ten patients contributed implants to more than one elevation group, and no patient contributed more than one implant to the same group. The demographic and clinical characteristics of the three elevation groups are summarized in Table 1. No statistically significant differences were detected among the groups in patient age, implant distribution according to patient sex, implant diameter, implant length, or implant site distribution (*P* > 0.05 for all). Baseline residual bone height differed significantly among the groups, being highest in the 3 mm group and lowest in the 5 mm group (*P* < 0.001). Given this baseline imbalance and the observed association between RBH and early FD change, baseline RBH was included as a covariate in the adjusted analyses.

**TABLE 1:** Demographic and clinical characteristics of the study sample by TSFE group.

Characteristic	3 mm	4 mm	5 mm	*p*
Implants, *n*	17	14	14	
Age, years (mean ± SD)	45.0 ± 9.9	44.0 ± 9.4	47.8 ± 9.2	0.554
Implants by patient sex, *n* (male/female)	10 / 7	6 / 8	6 / 8	0.583
Residual bone height, mm (mean ± SD)	6.65 ± 1.17	5.86 ± 1.03	5.00 ± 0.88	**<0.001**
Implant diameter, mm (mean ± SD)	4.31 ± 0.41	4.28 ± 0.37	4.40 ± 0.31	0.659
Implant length, mm (mean ± SD)	9.18 ± 1.01	9.14 ± 1.03	9.29 ± 0.99	0.926
Implant site, *n* (molar / premolar)	13 / 4	12 / 2	13 / 1	0.451

SD, standard deviation. Patient age is reported at the patient level. Sex distribution is reported at the implant level as the number of implants placed in male and female patients; therefore, the total corresponds to 45 implants rather than 35 unique patients. Implant diameter, implant length, residual bone height, and implant site are also reported at the implant level. *P*-values were obtained using one-way ANOVA for continuous variables and the Chi-square test for categorical variables.

According to the reliability analysis performed for the measurements in all three sinus elevation groups, the intraclass correlation coefficient (ICC) values indicated a high level of measurement reliability. In the 3 mm group, the ICC values were 0.788 for the apical region, 0.865 for the mesial region, and 0.817 for the distal region. In the 4 mm group, the ICC values were 0.856, 0.790, and 0.843 for the apical, mesial, and distal regions, respectively. In the 5 mm group, the corresponding ICC values were 0.878, 0.846, and 0.862.

No statistically significant difference was observed among the 3 mm, 4 mm, and 5 mm sinus elevation groups in terms of changes in trabecular bone structural complexity from baseline to postoperative 6 and 12 months (*P* > 0.05) (Table 2).

**TABLE 2:** Comparison of trabecular bone structural complexity changes among the 3 mm, 4 mm, and 5 mm TSFE groups from baseline to postoperative 6 and 12 months.

Time interval	3 mm	4 mm	5 mm	F	*p*
Baseline to 6 months	−0.001 ± 0.16	0.068 ± 0.14	0.014 ± 0.19	2.081	0.129
Baseline to 12 months	0.043 ± 0.14	0.008 ± 0.19	−0.012 ± 0.19	1.333	0.325

F: one-way analysis of variance (ANOVA). Values are expressed as the mean change in fractal dimension plus/minus standard deviation, where the change for each implant is the mean of the apical, mesial, and distal values.

**FIGURE 3:**
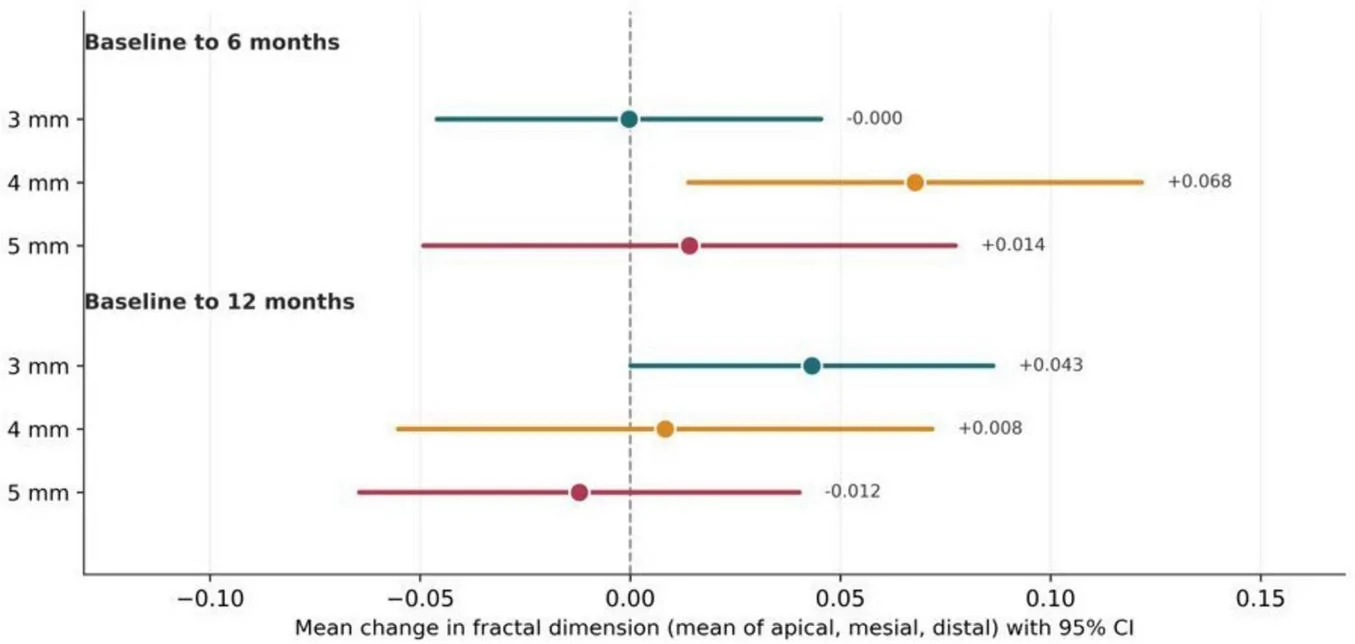
Between-group comparison of the mean change in fractal dimension with 95% confidence intervals. Each marker represents the group mean change and the horizontal bar the 95% confidence interval for the 3 mm, 4 mm, and 5 mm TSFE groups over the baseline-to-6-month and baseline-to-12-month intervals. The dashed vertical line denotes zero change. For each implant, the plotted change is the mean of the apical, mesial, and distal fractal dimension values. The overlapping confidence intervals, all of which include zero, are consistent with the non-significant between-group ANOVA results; the absence of a significant difference does not establish equivalence.

The absence of between-group differences is illustrated in Figure 3, in which the mean change in fractal dimension for each elevation group is displayed with its 95% confidence interval. For both the baseline-to-6-month and baseline-to-12-month intervals, all confidence intervals overlapped and crossed the line of no change, which is consistent with the non-significant between-group comparisons; this does not establish equivalence and does not exclude the possibility that the study was underpowered to detect small differences. When these between-group comparisons were repeated using linear mixed-effects models with patient as a random effect to account for within-patient clustering, the group effect remained non-significant for both the baseline-to-6-month (*P* = 0.247) and baseline-to-12-month (*P* = 0.332) intervals. Because the unadjusted between-group comparisons were already non-significant (for example, *P* = 0.129 for the baseline-to-6-month interval), these adjusted models are best regarded as sensitivity analyses. After adjustment for baseline residual bone height, the group effect remained non-significant for the baseline-to-6-month interval (ANCOVA *P* = 0.053; mixed-effects model *P* = 0.065), although the p-value moved closer to the conventional threshold, and for the baseline-to-12-month interval (ANCOVA *P* = 0.697). Baseline residual bone height was associated with early FD change (*P* = 0.029), indicating that the between-group findings should be interpreted cautiously.

The changes in FD values in the apical, mesial, and distal regions for each sinus elevation group at baseline, postoperative 6 months, and postoperative 12 months are presented in Table 3.

**TABLE 3:** Comparison of trabecular bone structural complexity by peri-implant region and time point across the different membrane elevation heights following TSFE.

Height	Time point	Apical	Mesial	Distal	F (p) between regions	F (p) over time
3 mm	Baseline	0.74 ± 0.14	0.73 ± 0.10	0.73 ± 0.11	F = 0.057, *p* = 0.945	**Apical:** F = 0.895, *p* = 0.418
	6 months	0.79 ± 0.12	0.75 ± 0.12	0.66 ± 0.13	F = 6.868, ***p* = 0.003**	**Mesial:** F = 1.039, *p* = 0.366
	12 months	0.76 ± 0.09	0.78 ± 0.15	0.78 ± 0.12	F = 0.189, *p* = 0.828	**Distal: F = 6.469, *p* = 0.004**
4 mm	Baseline	0.71 ± 0.09	0.76 ± 0.11	0.75 ± 0.13	F = 1.537, *p* = 0.234	**Apical:** F = 2.915, *p* = 0.072
	6 months	0.79 ± 0.10	0.80 ± 0.09	0.82 ± 0.13	F = 0.239, *p* = 0.789	**Mesial:** F = 2.622, *p* = 0.092
	12 months	0.76 ± 0.14	0.71 ± 0.12	0.78 ± 0.16	F = 1.043, *p* = 0.367	**Distal:** F = 0.953, *p* = 0.398
5 mm	Baseline	0.82 ± 0.12	0.77 ± 0.19	0.72 ± 0.14	F = 1.615, *p* = 0.220	**Apical:** F = 0.473, *p* = 0.629
	6 months	0.79 ± 0.09	0.74 ± 0.10	0.81 ± 0.12	F = 1.902, *p* = 0.169	**Mesial:** F = 0.115, *p* = 0.892
	12 months	0.79 ± 0.15	0.77 ± 0.15	0.71 ± 0.11	F = 1.472, *p* = 0.248	**Distal:** F = 2.197, *p* = 0.133

SD: standard deviation. Baseline: panoramic radiograph obtained on the day of implant placement. Postoperative: follow-up radiographs obtained at 6 and 12 months after implant placement. F (p) between regions: repeated-measures ANOVA comparing the apical, mesial, and distal regions (paired within-implant measurements) at each time point. F (p) over time: repeated-measures ANOVA within each region over time. Values in bold red indicate statistical significance (*p* < 0.05).

**FIGURE 4:**
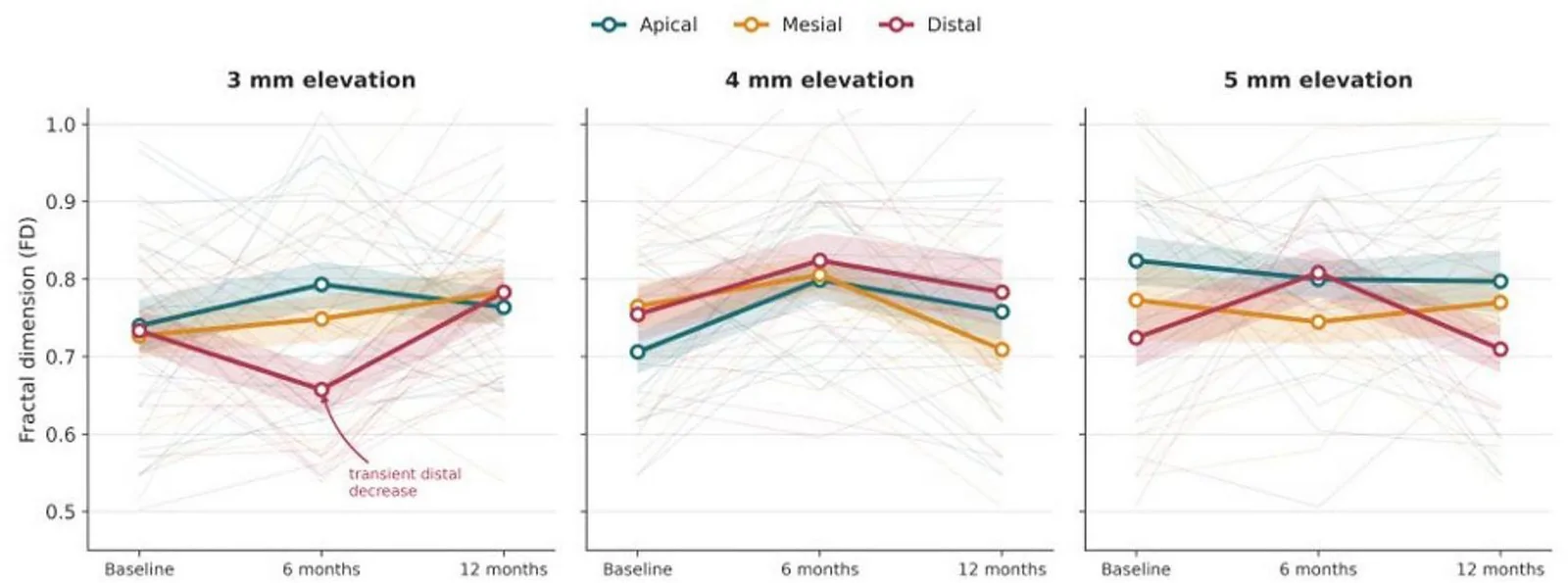
Individual and mean fractal dimension trajectories by region for each TSFE group. Faint lines represent single-implant trajectories; bold lines with markers represent the regional means, and shaded bands the standard error. The transient decrease in distal FD values at 6 months in the 3 mm group is annotated.

The region- and time-dependent trajectories underlying Table 3 are shown in Figure 4, where individual implant profiles are overlaid with the regional mean course for each elevation height. In the 4 mm and 5 mm groups, the regional means showed relatively limited variation across time.

**FIGURE 5:**
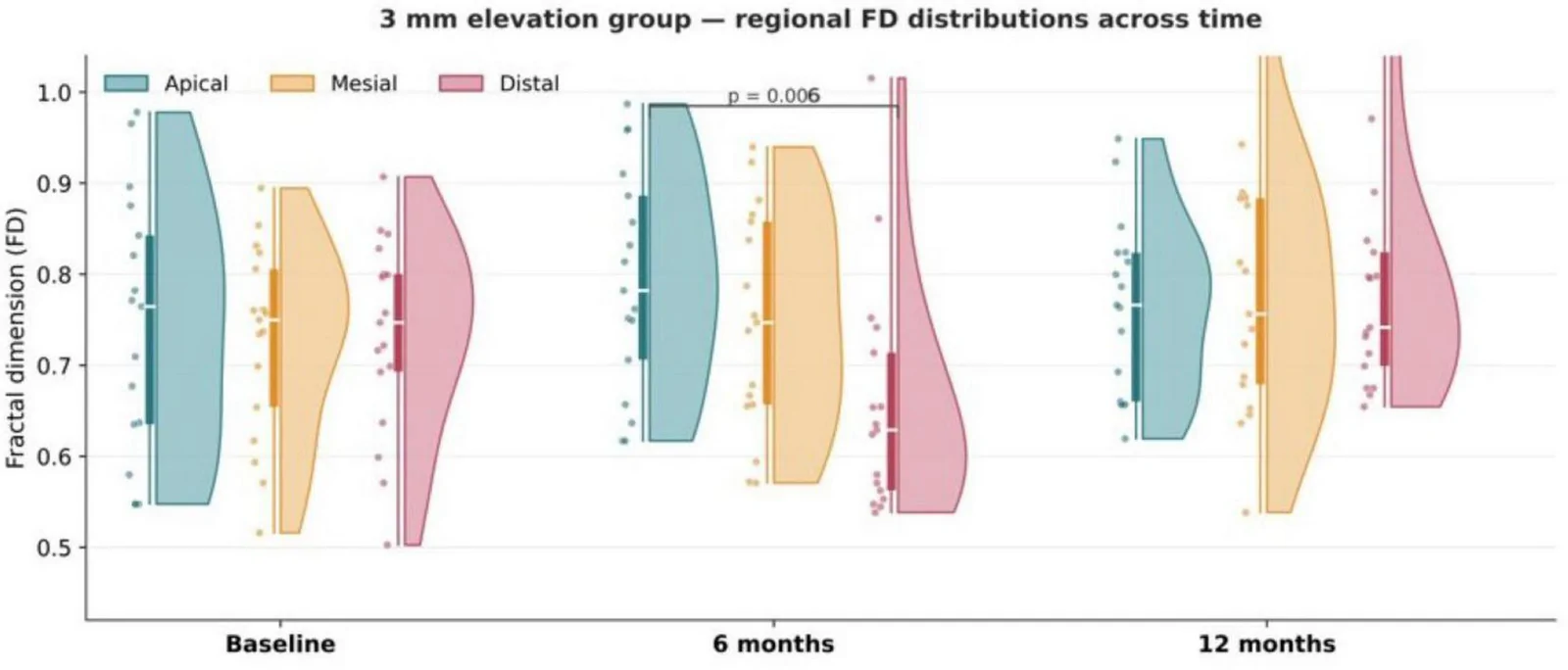
Distribution of regional fractal dimension values in the 3 mm TSFE group across time. Raincloud representation combining the smoothed density (half-violin), the interquartile box with median, and individual measurements for the apical, mesial, and distal regions at baseline, 6 months, and 12 months. The bracket marks the significant apical-versus-distal difference at 6 months.

In the 3 mm sinus elevation group, no significant difference was observed between baseline and the 12-month follow-up in terms of FD values (*P* > 0.05). At postoperative 6 months, the mean FD values were 0.79 plus/minus 0.12 in the apical region, 0.75 plus/minus 0.12 in the mesial region, and 0.66 plus/minus 0.13 in the distal region. A statistically significant difference was observed among the regions at this time point (F = 6.868, *P* = 0.003). No significant time-dependent change was detected in the apical or mesial regions (*P* > 0.05). In contrast, the FD value in the distal region changed significantly over time (F = 6.469, *P* = 0.004). The regional difference at 6 months remained significant in the nonparametric analysis (Friedman *P* = 0.007); the apical-versus-distal comparison was significant in the Wilcoxon signed-rank test (unadjusted *P* = 0.002; Bonferroni-adjusted *P* = 0.006). All four of these comparisons remained significant after Benjamini-Hochberg correction for the false discovery rate (adjusted *P* ≤ 0.007). The regional distributions for the 3 mm group are shown in Figure 5.

At postoperative 6 months, a statistically significant difference in FD values was found between the apical and distal regions (*P* = 0.006) (Table 4).

**TABLE 4:** Pairwise comparison of regional trabecular bone structural complexity in the 3 mm TSFE group at postoperative 6 months.

Comparison (6 months)	*p* *
Apical vs. Mesial	0.500
Apical vs. Distal	**0.006**
Mesial vs. Distal	0.154

*The significant apical-versus-distal difference was confirmed by the Wilcoxon signed-rank test (unadjusted *P* = 0.002; Bonferroni-adjusted *P* = 0.006). Value in bold red indicates statistical significance.

In the distal region of the 3 mm group, where the time effect was significant, *post-hoc* analysis revealed a statistically significant difference between postoperative 6 and 12 months (*P* = 0.013) (Table 5). This difference remained significant on nonparametric testing (Wilcoxon signed-rank *P* = 0.006).

**TABLE 5:** Pairwise comparison of the effect of time on trabecular bone structural complexity in the distal region of the 3 mm TSFE group.

3 mm distal region	*p* *
Baseline vs. 6 months	0.241
Baseline vs. 12 months	0.187
6 months vs. 12 months	**0.013**

*p-values adjusted using Bonferroni correction. Value in bold red indicates statistical significance.

**FIGURE 6:**
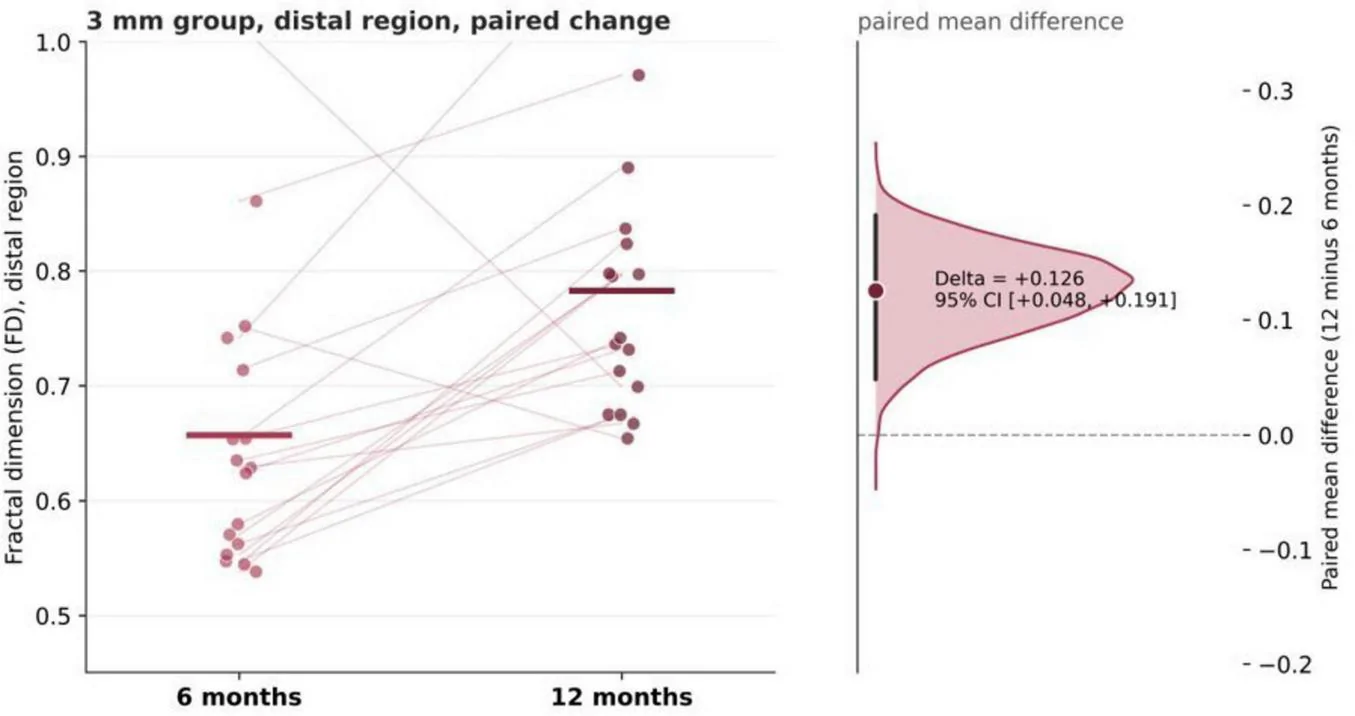
Paired estimation plot of the distal fractal dimension change in the 3 mm TSFE group between 6 and 12 months. The left axis shows paired individual values with group means; the right axis shows the bootstrap distribution of the paired mean difference (12 minus 6 months) with its 95% confidence interval. The dashed line marks no difference.

The magnitude of this recovery is shown in Figure 6. The paired mean difference between the 6- and 12-month distal FD values was +0.13 (95% confidence interval, 0.05 to 0.19), with the confidence interval excluding zero.

In the 4 mm sinus elevation group, no significant difference was observed among baseline, postoperative 6 months, and postoperative 12 months in terms of FD values (*P* > 0.05). Similarly, no significant time-dependent change was detected in the apical, mesial, or distal regions (*P* > 0.05) (Table 3). In the 5 mm sinus elevation group, no statistically significant difference was found among baseline, postoperative 6 months, and postoperative 12 months in terms of FD values. In addition, no significant interregional or intraregional differences were observed in the apical, mesial, or distal regions (*P* > 0.05) (Table 3).

## Discussion

Studies evaluating peri-implant trabecular bone structural complexity using fractal analysis after TSFE at different membrane elevation heights are limited. In this study, trabecular bone structural complexity in the apical, mesial, and distal regions around implants with 3 mm, 4 mm, and 5 mm sinus membrane elevation was evaluated using fractal dimension (FD) analysis on panoramic radiographs. Measurements were performed at baseline and at postoperative 6 and 12 months. The main finding was that no statistically significant difference was observed among the 3 mm, 4 mm, and 5 mm groups in terms of FD changes from baseline to 6 or 12 months. Within the limitations of this study, this finding indicates only that no between-group difference was detected in the present sample and should not be interpreted as evidence of an equivalent trabecular response or an independent effect of elevation height.

In the posterior maxilla, maxillary sinus elevation is widely used to enable implant-supported rehabilitation in cases with insufficient bone volume (1). TSFE is less invasive than the lateral window approach and can be performed simultaneously with implant placement in appropriate cases (19). However, because the surgical field cannot be directly visualized, intraoperative assessment of membrane integrity may be more difficult (20). In addition, membrane elevation height and residual ridge height are important factors associated with Schneiderian membrane perforation (21). Therefore, evaluating peri-implant bone response after TSFE, particularly at elevations greater than 3 mm, is clinically relevant.

No statistically significant between-group differences in FD change were detected among the 3 mm, 4 mm, and 5 mm groups. Previous studies have reported predictable clinical outcomes with the TSFE approach in the posterior maxilla with limited bone volume (19, 22). Nevertheless, in cases requiring greater elevation, anatomical factors, membrane elasticity, and perforation risk should be carefully considered (23, 24). In the present study, no statistically significant regional or temporal FD changes were detected in the 4 mm and 5 mm groups. This finding should not be interpreted as evidence of biological stability or a favorable effect of the procedure.

Fractal analysis is a non-invasive method for quantitatively evaluating the geometric complexity of trabecular bone (25). Previous studies have shown that peri-implant trabecular bone can be assessed by fractal analysis on panoramic radiographs in apical, mesial, and distal regions (26). It has also been reported that bone remodeling after maxillary sinus floor elevation can be monitored using FD analysis; however, FD values should not be considered equivalent to histomorphometric bone volume (14, 27). Unlike earlier reports that assessed ossification after lateral window sinus floor elevation, such as the study by Sancar et al. (17), the present work focused on the TSFE approach with simultaneous implant placement and compared different membrane elevation heights; this difference in surgical approach and design limits direct comparison and underlines the specific contribution of the present study to the TSFE literature. In addition, unlike Akpinar et al. (26), who compared fractal dimension between lateral window sinus floor elevation and TSFE and reported differences between techniques, the present study focused exclusively on TSFE and compared different elevation heights; and whereas Mu et al. (27) examined fractal dimension changes after prosthetic loading, the present study evaluated baseline, 6-month, and 12-month follow-up, with definitive prosthetic loading occurring between 6 and 9 months, and did not detect statistically significant temporal FD changes in the 4 mm and 5 mm groups. These differences in design and timing may partly explain the varied findings across studies and underscore the need for standardized fractal analysis protocols. Therefore, the present findings should be interpreted as changes in radiographic trabecular structural complexity rather than direct evidence of histological bone formation or bone mineral density.

The most notable regional finding was observed in the 3 mm group, where the distal radiographic structural complexity at 6 months was lower than the apical value and then increased between 6 and 12 months (paired mean difference +0.13; 95% confidence interval excluding zero). It should be emphasized that fractal dimension reflects radiographic textural complexity rather than histological bone formation, so this pattern describes a change in radiographic trabecular organization and not a directly observed biological event. Any biological account, such as transiently slower trabecular organization followed by later adaptation, remains speculative. Several explanations may be considered, none of which can be confirmed here: a region-specific response related to the osteotomy or drilling path, a healing-phase effect around the 6-month remodeling window, or, given the small size of this subgroup, a chance fluctuation or statistical artifact. Notably, the 3 mm group had the highest baseline residual bone height, so a purely loading-based explanation would be counterintuitive; this further reinforces the need for cautious, hypothesis-generating interpretation. Because occlusal forces, functional load distribution, and prosthetic loading patterns were not directly measured, these interpretations should be regarded as hypotheses requiring prospective confirmation.

In the 4 mm and 5 mm groups, no statistically significant regional or temporal FD changes were detected during follow-up. Previous peri-implant studies have suggested that FD analysis may be useful for monitoring changes in radiographic trabecular structural complexity after loading (28–31). Nevertheless, the absence of statistically significant FD changes should not be interpreted as evidence of a homogeneous trabecular response, histological stability, or absence of biological remodeling.

This study contributes to the literature by evaluating both regional and time-dependent changes in peri-implant trabecular bone structural complexity after transcrestal sinus floor elevation with different membrane elevation heights. Previous studies have used FD analysis to evaluate changes in peri-implant trabecular bone (32). More recently, Sozen et al. (33) used CBCT-based FD analysis to evaluate graft osteogenesis following maxillary sinus floor elevation surgery. Their study assessed grafted regions using CBCT, whereas the present study evaluated peri-implant trabecular bone structural complexity after graft-free transcrestal sinus floor elevation using panoramic radiographs. Therefore, differences in graft use, imaging modality, and ROI definition limit direct comparison between the studies. From a radiographic standpoint, no statistically significant regional or temporal FD changes were detected in the 4 mm and 5 mm groups. However, FD analysis does not directly measure load distribution, implant stability, implant survival, or biomechanical performance; therefore, no conclusions regarding these clinical or biomechanical outcomes can be drawn from the present findings.

This study has several limitations. First, it had a retrospective design and a relatively limited sample size; accordingly, the non-significant between-group results are best interpreted together with the confidence intervals reported in Figure 3 rather than as proof of equivalence. In addition, several patients contributed more than one implant; to address the resulting within-patient clustering, the primary between-group comparisons were repeated using mixed-effects models with patient as a random effect, and the conclusions were unchanged, although the sample was not large enough to support fully adjusted multilevel modeling of every regional and temporal subgroup. Furthermore, baseline residual bone height differed significantly among the elevation groups and was associated with early FD change. Although RBH-adjusted analyses were performed, the independent contribution of elevation height cannot be isolated, and residual confounding cannot be excluded. Moreover, the *a priori* sample size calculation assumed a large effect size (f = 0.60), which is larger than would be expected for subtle changes in trabecular architecture; the study was therefore likely underpowered to detect small between-group differences, and the non-significant findings should be interpreted with corresponding caution. Second, the evaluations were performed only on panoramic radiographs. Although panoramic radiographs are commonly used in clinical practice, their two-dimensional nature, magnification differences, and resolution limitations may restrict detailed assessment of trabecular bone structure. Third, FD values were not compared with histomorphometric data or CBCT-based volumetric measurements. Therefore, FD should not be interpreted as a direct indicator of bone volume, bone mineral density, or histological bone formation. In addition, implant design, primary stability, prosthetic loading time, occlusal force distribution, and patient-related biological factors may influence peri-implant bone remodeling. Future prospective studies with larger samples and three-dimensional imaging methods should evaluate these variables.

## Conclusion

This study demonstrated that peri-implant trabecular bone structural complexity can be non-invasively evaluated using fractal dimension analysis after TSFE. No statistically significant differences were observed among the 3 mm, 4 mm, and 5 mm elevation groups in FD changes from baseline to postoperative 6 and 12 months. Because non-significance does not establish equivalence and baseline RBH differed among the groups, these findings should be interpreted as preliminary group-level associations rather than evidence of an independent effect of elevation height. Larger prospective studies with formal equivalence testing are needed before definitive conclusions can be drawn.

In the 3 mm group, a transient decrease in distal FD values at 6 months followed by an increase at 12 months was observed; this should be interpreted cautiously as a region-specific, hypothesis-generating change in radiographic structural complexity rather than a confirmed biological event. Overall, fractal analysis may serve as a useful non-invasive radiographic method for monitoring peri-implant trabecular structural complexity after TSFE.

## Data Availability

The raw data supporting the conclusions of this article will be made available by the authors upon reasonable request, in accordance with applicable ethical and institutional restrictions.

## References

[1] Danesh-Sani S Loomer P Wallace SS. A comprehensive clinical review of maxillary sinus floor elevation: anatomy, techniques, biomaterials and complications. *Br J Oral Maxillofac Surg.* (2016) 54:724–30. 10.1016/j.bjoms.2016.05.008 27235382

[2] Araújo M Lindhe J. Dimensional ridge alterations following tooth extraction. An experimental study in the dog. *J Clin Periodontol.* (2005) 32:212–8. 10.1111/j.1600-051X.2005.00642.x 15691354

[3] Lim H Kim S Kim D Herr Y Chung J Shin S. Factors affecting maxillary sinus pneumatization following posterior maxillary tooth extraction. *J Periodontal Implant Sci.* (2021) 51:285–95. 10.5051/jpis.2007220361 34387048 PMC8367647

[4] Tatum H. Maxillary and sinus implant reconstructions. *Dent Clin North Am.* (1986) 30:207–29. 10.1016/S0011-8532(22)02107-3 3516738

[5] Boyne P. Grafting of the maxillary sinus floor with autogenous marrow and bone. *J Oral Surg.* (1980) 38:613–6. 6993637

[6] Alshamrani A Mubarki M Alsager A Alsharif H AlHumaidan S Al-Omar A et al. Maxillary sinus lift procedures: an overview of current techniques, presurgical evaluation, and complications. *Cureus.* (2023) 15:e49553. 10.7759/cureus.49553 38156177 PMC10753870

[7] Summers R. A new concept in maxillary implant surgery: the osteotome technique. *Compendium.* (1994) 15:152–62. 8055503

[8] Fermergård R Ăstrand P. Osteotome sinus floor elevation and simultaneous placement of implants–a 1-year retrospective study with Astra Tech implants. *Clin Implant Dent Relat Res.* (2008) 10:62–9. 10.1111/j.1708-8208.2007.00062.x 18254742

[9] Kadkhodazadeh M Alimardani Y Azadi A Daneshvar A Amid R Khaleghi A. Clinical outcomes of implants placed with transcrestal maxillary sinus elevation: a systematic review and meta-analysis. *Br J Oral Maxillofac Surg.* (2024) 62:685–703. 10.1016/j.bjoms.2024.05.006 39098575

[10] Gargallo-Albiol J Tattan M Sinjab K Chan H Wang H. Schneiderian membrane perforation via transcrestal sinus floor elevation: a randomized ex vivo study with endoscopic validation. *Clin Oral Implants Res.* (2019) 30:11–9. 10.1111/clr.13388 30450593

[11] Boyacıgil D Er N Karaca Ç Koç O. The effect of residual bone height and membrane thickness on sinus membrane perforation in crestal sinus grafting: a prospective clinical study. *Int J Oral Maxillofac Surg.* (2021) 50:251–7. 10.1016/j.ijom.2020.05.018 32600745

[12] Mandelbrot B. *The Fractal Geometry of Nature.* New York: Freeman (1982).

[13] Zeytinoğlu M İlhan B Dündar N Boyacioğlu H. Fractal analysis for the assessment of trabecular peri-implant alveolar bone using panoramic radiographs. *Clin Oral Investig.* (2015) 19:519–24. 10.1007/s00784-014-1245-y 24802628

[14] da Silva M Dos Santos H Ruhland L Rabelo G Badaró M. Fractal analysis of dental periapical radiographs: a revised image processing method. *Oral Surg Oral Med Oral Pathol Oral Radiol.* (2023) 135:669–77. 10.1016/j.oooo.2022.11.014 36609053

[15] de Molon R de Paula W Spin-Neto R Verzola M Tosoni G Lia R et al. Correlation of fractal dimension with histomorphometry in maxillary sinus lifting using autogenous bone graft. *Braz Dent J.* (2015) 26:11–8. 10.1590/0103-6440201300290 25672378

[16] Şimşek M Alan H Dedeoğlu N. A fractal evaluation study of implant-supported overdentures. *Oral Surg Oral Med Oral Pathol Oral Radiol.* (2025) 140:264–70. 10.1016/j.oooo.2025.01.730 40268666

[17] Sancar B Dedeoglu N Cetiner Y Aral C Altun O. Using the fractal dimension method to assess ossification after open sinus lift surgery. *J Health Sci Med.* (2022) 5:326–30. 10.32322/jhsm.1027651

[18] White S Rudolph D. Alterations of the trabecular pattern of the jaws in patients with osteoporosis. *Oral Surg Oral Med Oral Pathol Oral Radiol Endod.* (1999) 88:628–35. 10.1016/s1079-2104(99)70097-1 10556761

[19] Valentini P Stacchi C. Prevention and management of intra-operative complications in maxillary sinus augmentation: a review. *Clin Implant Dent Relat Res.* (2025) 27:e13397. 10.1111/cid.13397 39379340 PMC11789845

[20] Lyu M Xu D Zhang X Yuan Q. Maxillary sinus floor augmentation: a review of current evidence on anatomical factors and a decision tree. *Int J Oral Sci.* (2023) 15:41. 10.1038/s41368-023-00248-x 37714889 PMC10504247

[21] Deping C Qian L Xuemin P Hong P Bin R Yucheng S. Clinical procedure of transalveolar technique for sinus floor elevation. *Chin J Oral Implantol.* (2023) 28:128. 10.12337/zgkqzzxzz.2023.04.011

[22] Xue H Wen J Liu C Shuai X Zhang X Kang N. Modified transcrestal sinus floor elevation with concomitant implant placement in edentulous posterior maxillae with residual bone height of 5 mm or less: a non-controlled prospective study. *Int J Oral Maxillofac Surg.* (2023) 52:495–502. 10.1016/j.ijom.2022.08.014 36058822

[23] Lee C Choksi K Shih M Rosen P Ninneman S Hsu Y. The impact of sinus floor elevation techniques on sinus membrane perforation: a systematic review and network meta-analysis. *Int J Oral Maxillofac Implants.* (2023) 38:681–96. 10.11607/jomi.10048 37669518

[24] Mazor Z Gaspar J Silva R Pohl S Gandhi Y Huwais S et al. Maxillary sinus membrane perforation rate utilizing osseodensification-mediated transcrestal sinus floor elevation: a multicenter clinical study. *Clin Implant Dent Relat Res.* (2024) 26:1172–80. 10.1111/cid.13368 39187444 PMC11660531

[25] Wen C Zhang Q. Pilot study of fractal dimension analysis of osteogenesis for bone substitute materials of Bio-Oss in lateral sinus augmentation. *PLoS One.* (2023) 18:e0296248. 10.1371/journal.pone.0296248 38157335 PMC10756558

[26] Akpınar H Ozbey F Yıldırım B. Comparison of trabecular bone structure using fractal dimension analysis in patients undergoing lateral window and transcrestal sinus lift procedures: a retrospective cohort study. *BMC Med Imaging.* (2025) 25:430. 10.1186/s12880-025-01976-8 41152752 PMC12570708

[27] Mu T Lee D Park K Moon I. Changes in the fractal dimension of peri-implant trabecular bone after loading: a retrospective study. *J Periodontal Implant Sci.* (2013) 43:209–14. 10.5051/jpis.2013.43.5.209 24236242 PMC3825987

[28] Torul D Yilmaz M Erzurumlu Z Omezli M. Evaluation of changes in peri-implant bone architecture at different stages of implant treatment with fractal analysis. *J Stomatol Oral Maxillofac Surg.* (2025) 126:102508. 10.1016/j.jormas.2025.102508 40714258

[29] Dundar N Aslan E Mutlu O. Fractal dimension, lacunarity, and bone area fraction analysis of peri-implant trabecular bone after prosthodontic loading. *Oral Radiol.* (2025) 41:120–30. 10.1007/s11282-024-00784-0 39523226

[30] Ustaoglu G Bulut D Aydin Z. The effect of single-tooth implant restorations on prognosis of adjacent teeth and on fractal dimension of peri-implant trabecular bone: a retrospective study. *Selcuk Dent J.* (2022) 9:208–15.

[31] Yurtoglu N Tozum T Uysal S. Evaluation of peri-implant bone changes with fractal analysis. *J Clin Med.* (2025) 14:3820. 10.3390/jcm14113820 40507583 PMC12156791

[32] Guarnieri R Miccoli G Di Nardo D D’Angelo M Morese A Seracchiani M et al. Effect of a laser-ablated micron-scale modification of dental implant collar surface on changes in the vertical and fractal dimensions of peri-implant trabecular bone. *Clin Ter.* (2020) 171:e385–92. 10.7417/CT.2020.2245 32901779

[33] Sozen E Aytugar E Ertas E Cene E Kara M. Evaluation of graft osteogenesis using fractal dimension analysis on cone-beam computed tomography images following maxillary sinus lift surgery. *BMC Oral Health.* (2025) 25:1346. 10.1186/s12903-025-06695-8 40841897 PMC12369246

